# Regulatory Components for Bacterial Cell-Free Systems Engineering

**DOI:** 10.1021/acssynbio.4c00574

**Published:** 2024-11-07

**Authors:** Pao-Wan Lee, Sebastian J. Maerkl

**Affiliations:** Institute of Bioengineering, School of Engineering, École Polytechnique Fédérale de Lausanne, Lausanne 1015, Switzerland

**Keywords:** Synthetic biology, Gene regulatory network, Cell-free Systems, Gene circuits

## Abstract

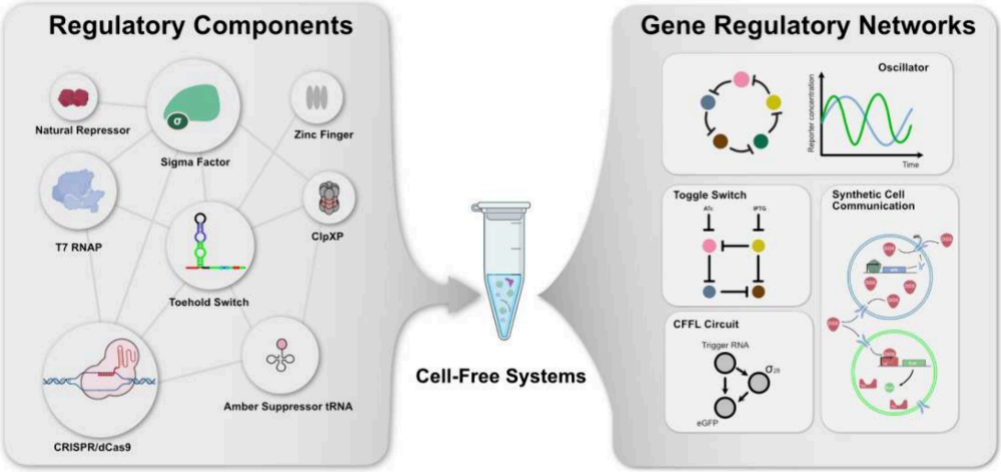

Cell-free systems are advancing synthetic biology through fast prototyping and modularity. Complex regulatory networks can now be implemented in cell-free systems enabling various applications, such as diagnostic tool development, gene circuit prototyping, and metabolic engineering. As functional complexity increases, the need for regulatory components also grows. This review provides a comprehensive overview of native as well as engineered regulatory components and their use in bacterial cell-free systems.

## 1 Introduction

Cell-free systems (CFSs) are capable of transcription and translation in an *in vitro* environment. Engineering CFSs offer several advantages over living systems. For example CFSs can express toxic proteins that are otherwise challenging to express in cellular hosts. The use of linear DNA templates in the CFSs reduces the time required for iterating through the design-build-test cycle, by eliminating the time-consuming plasmid cloning and transformation process.^1^ Moreover, the absence of cell membranes in cell-free reactions allows for easy manipulation and addition of components. Transcriptional levels can also be easily and precisely controlled by directly altering input DNA concentrations. Furthermore, molecules can be directly added to the reaction as well, including molecules that normally do not traverse the cell membrane or cell wall, which is particularly useful in biosensing applications.^2^ The CFS’s open and modular nature therefore simplifies the engineering process of prototyping gene expression^3^ and enables applications that would be difficult to implement using cellular engineering.

Two classes of CFSs exist today: lysate-based systems and the reconstituted PURE (protein synthesis using recombinant elements) system. Lysate-based CFSs are produced through lysis of prokaryotic or eukaryotic cells. The most commonly used lysate-based CFS is based on *Escherichia coli (E. coli)*, and the process has been optimized to become more affordable and efficient and can now be easily produced.^4^ The protein production yield of *E. coli* lysates has been gradually improved over the years and it was recently shown that an *E. coli* lysate can produce 8 mg/mL of eGFP.^5^ The other commonly used CFS is PURE, developed by Shimizu and Ueda in 2001.^6^ PURE contains 36 purified proteins required for transcription and translation as well as ribosomes, tRNAs, and an energy solution. Due to its defined nature, PURE is an appealing chassis for cell-free systems engineering. Although PURE is commercially available from various suppliers, the cost of PURE remains high and making the PURE system in house requires the cumbersome purification of 36 proteins and ribosomes. To drastically simplify this process we recently developed the OnePot PURE method, which expresses and purifies all 36 nonribosomal proteins in a single step.^7,8^ The nonribosomal protein content of the PURE system was also recently optimized toward self-regeneration, showing that the PURE system can be highly diluted while retaining protein synthesis capacity, and the addition of crowding agents to dilute PURE formulations increased protein synthesis rates and yield.^9^ The distinction between lysate-based and PURE systems has been previously discussed in more detail.^10^

Gene regulatory networks (GRNs) are one of the major cellular control systems to process and propagate information. GRNs allow cells to respond to internal and external signals or perturbations and provide adaptability and systems robustness. Synthetic biology makes use of components used by living systems to de novo engineer synthetic GRNs that achieve specific functions. Notable circuits are the toggle switch,^11^ the repressilator^12^ and a tunable oscillator.^13^ Brophy et al. summarized gene circuits constructed with regulatory components in cells, including oscillators, pulse generators, logic gates, bistable switches, adders and memory circuits.^14^

However, cells are complex systems composed of many noncharacterized components and a poorly defined environment that give rise to difficulties in engineering increasingly complex systems *in vivo*. Unlike cellular systems, the composition of a CFS such as PURE is well-defined, and this defined nature makes the CFS more amenable to mathematical modeling which is often beneficial for designing complex synthetic systems. Several computational models for the CFS have been built that include biochemical reactions.^15−17^ The computational model built by Shimizu’s group to simulate the PURE system included hundreds of mass-action reactions to predict expression of peptides composed of 2 amino acids.^18^ Although considerable work is still required in this area, models of the PURE system are significantly more tractable than comparable whole-cell models.^19,20^

Starting in the early 2000s, GRNs began being implemented in cell-free lysate systems.^21^ Noireaux et al. demonstrated the implementation of regulatory components to achieve protein expression cascades, in which different RNA polymerases (RNAPs) are produced sequentially, and transcribe downstream genes regulated by different promoters. In the past two decades, more and more circuits have been implemented in CFSs, such as oscillators,^22,23^ toggle switches,^1,24^ logic gates,^3^ systems for molecular diagnostics,^25−27^ noise filters,^28^ and synthetic cell communication.^29,30^ However, similar to cellular systems the functional complexity of the circuits implemented in cell-free environments is limited by the available regulatory components. To build cell-free regulatory networks with increased functionality, two main problems need to be solved. First, we need to develop additional programmable and orthogonal regulatory components. Second, we must quantitatively characterize the regulatory components and develop computational models to improve circuit design predictability.

The use of CFSs facilitates the quantitative characterization of cell-free regulatory components by both speeding up the characterization process and permitting quantitative characterization to be performed more easily than in cellular systems. We previously utilized high-throughput microfluidics in combination with cell-free protein synthesis to synthesize a large library of Zinc-finger transcription factors(TFs) and characterize their binding specificities in a large-scale experiment.^31^ The functional characteristics of these zinc finger TFs and promoters were then characterized in a cell-free gene regulatory system, allowing predictive design and implementation of complex logic gates.^3^ These types of quantitative studies are now possible through the combination of CFSs and highly integrated microfluidic devices.

**Figure 1:**
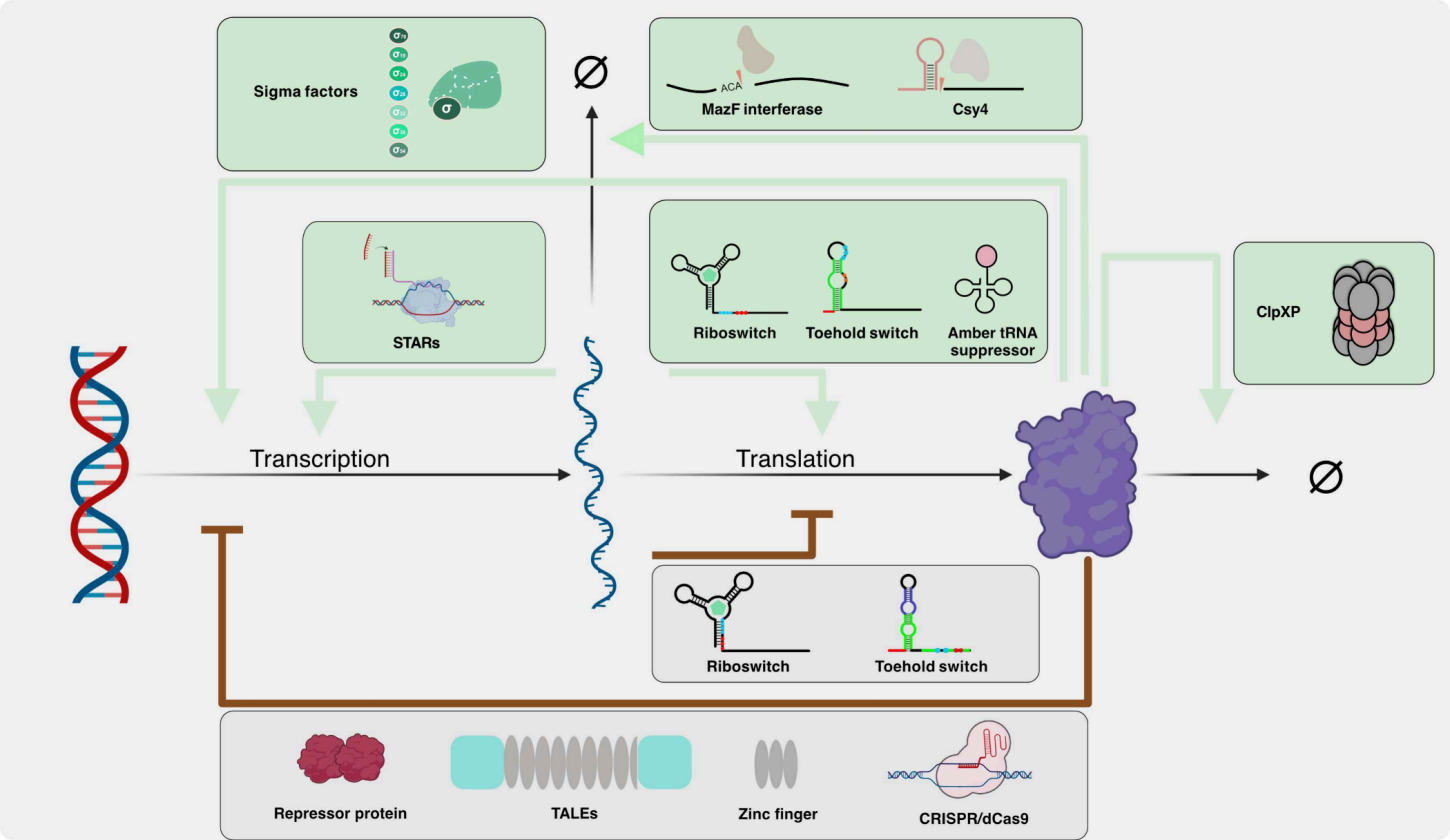
Cell-free regulatory components toolbox. RNA and protein components regulate gene expression at various stages, including transcription, translation, RNA degradation, and protein degradation.

This review paper discusses components that have been characterized or applied in *E. coli*-based lysate and PURE systems. We discuss a broad range of regulators that allow control over various central processes including transcription, translation, RNA degradation, and protein degradation (Figure 1). Table 1 provides a list of the components discussed, along with the respective CFS in which each has been implemented. This article aims to summarize the current toolbox available to researchers to build genetic networks in CFSs. Additionally, the article covers regulatory components developed in living systems that have the potential to be applied in CFSs, thus further expanding the toolbox for cell-free circuit design in future.

**Table 1:** Table of Gene Regulator Components^a^

Table of gene regulator components in the articles			
Name	Regulation components	Cell-free system	Reference
Sigma factor	Sigma factor	*E. coli* lysate (L)	(5)
Synthetic Zinc finger protein	Zinc finger protein	*E. coli* lysate (L)	(3)
TALE repressor	TALE protein	PUREfrex2.0 (P)	(32)
Lac repressor	LacI protein	Qiagen EasyXpres (L)	(33)
TetR repressor	TetR protein	Qiagen EasyXpres (L)	(33)
CI repressor	CI protein	*E. coli* lysate (L)	(34)
STARs	Activation RNA sequence	My TX-TL *E. coli* Lysate (L)	(35)
CRISPR/dCas9	CRISPR/dCas9 protein, Guide RNA	*E. coli* lysate (L)	(36)
CRISPRa	CRISPR/dCas9, Guide RNA, engineered SoxS	*E. coli* lysate (L)	(37)
iiT7 RNAP	Zinc finger T7 RNAP fusion	N.A.	(38)
Split T7 RNAP	2 split T7 RNAPs	N.A.	(39)
Fragmented T7 RNAP	4 fragmented T7 RNAPs	N.A.	(40)
Opto T7RNAP	Mag-T7 RNAP fusion	N.A.	(41)
Riboswitch	Riboswitch RNA, target molecules	PURE, *E. coli* lysate (L)	(42)
Toehold switch	Regulated RNA, activator or repressor RNA	PURExpress (P), *E. coli* lysate (L)	(43)(44)
MazF interferase	MazF protein	*E. coli* lysate (L)	(45)
Cys4 Ribonuclease	Cys4 Protein	*E. coli* lysate (L)	(36)
Amber suppressor tRNA	Amber suppressor tRNA	PURExpress (P)	(22)
ClpXP	ClpXP, tagged protein	PURExpress (P)	(22)

a The table lists components that have been applied in cell-free lysate based (L) and PURE (P) based systems. Regulatory components have not been applied in CFS but have the potential to be implemented are also included in the table, which is labeled as (N.A.).

## 2 Transcriptional Regulation

### 2.1 Sigma Factors

**Figure 2:**
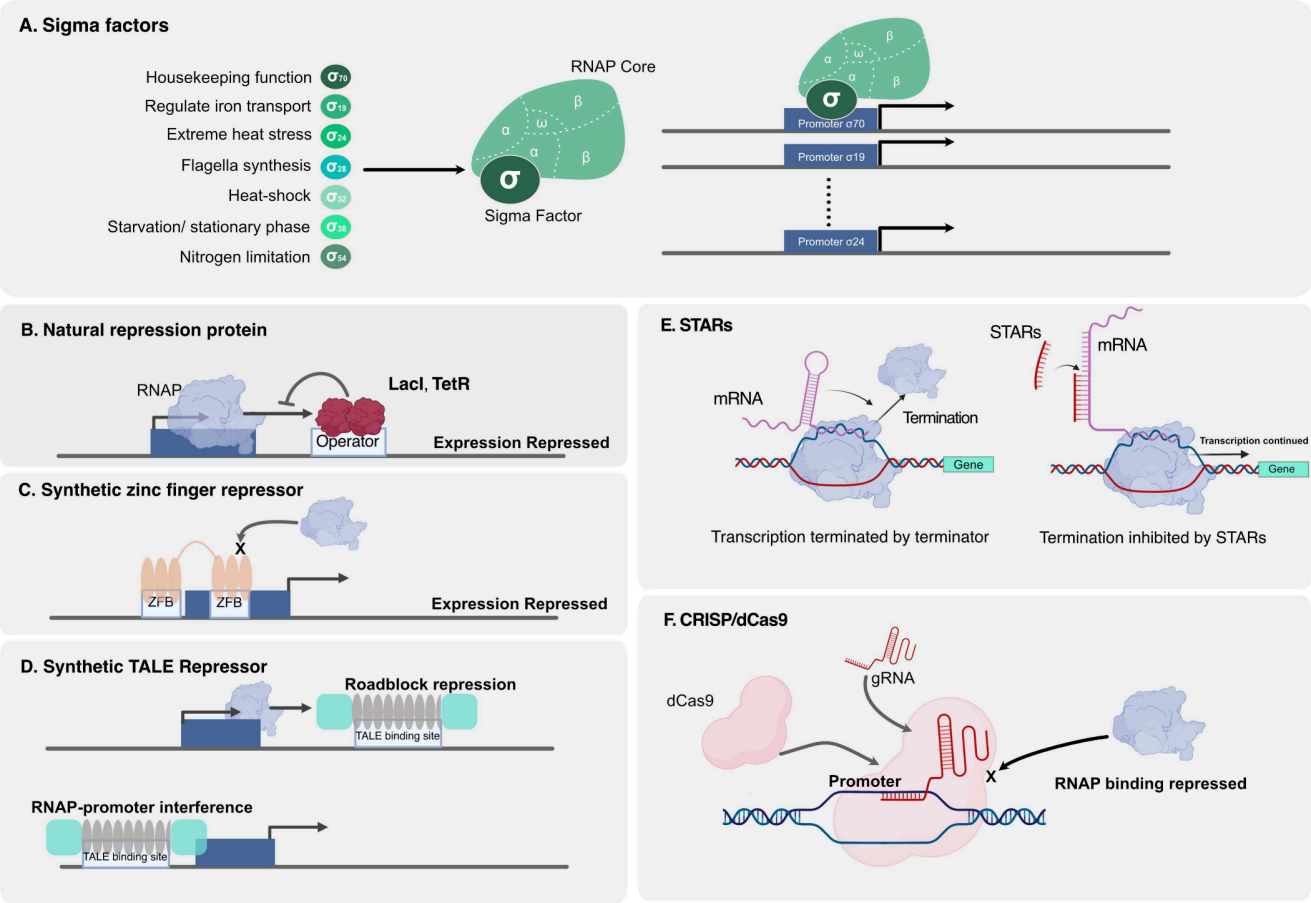
Mechanism of transcriptional regulatory components. (A) Sigma factors recognize and bind to different promoters under various conditions. Examples include seven sigma factors involved in different functions.^5^ (B) Natural transcriptional repressors like LacI and TetR bind to operator regions near the promoter, preventing RNAP from transcribing the downstream genes to repress gene expression.^1,33,46^ (C) Synthetic zinc finger repressor designed to bind target DNA sequences close to promoter region, thereby blocking RNAP and repressing transcription.^3,31^ (D) Synthetic TALE repressors function in two ways. First, through roadblock repression, where they obstruct RNAP. Second, through interfering directly with RNAP-promoter interactions by binding right upstream of the promoter.^32^ (E) STARs prevent transcription termination, enabling the continuation of transcription by interfering with terminator sequences.^35^ (F) CRISPR/dCas9 System utilizes dCas9 protein guided by a specific gRNA to bind to promoter regions, effectively repressing transcription by blocking RNAP binding.^36,37^

In the *E. coli* lysate CFS, transcription is carried out by the native RNAP, which consists of 6 subunits: two α, two β, ω, and σ factor (Figure 2A). The σ factor is responsible for recognizing specific promoter sequences to initiate transcription. Different σ factors recognize different promoter sequences and transcribe the corresponding genes. *E. coli* uses σ factors to control transcription of different genetic programs in response to varying growth conditions. For example, σ32 is responsible for producing heat shock proteins and is only produced under stress conditions. In the cell-free toolbox 3.0,^5^ seven different sigma factors were integrated into the CFS, allowing independent control of different promoters. Researchers can express genes activated by specific sigma factors with different strengths, which have been characterized in the system.

### 2.2 Natural Transcriptional Repressors

Repressor proteins such as LacI and TetR are frequently employed in *E. coli* to regulate gene expression (Figure 2B). These repressors endow cells with the capacity to respond to molecular signals. Several TFs that can repress transcription have been tested in CFSs. LacI is commonly used to create an inducible plasmid construct where the gene is expressed only under inducing conditions. It has been applied in CFSs to repress the T7 promoter.^1,33,46^ Initial efforts showed that adding a LacI binding site downstream of the T7 promoter did not significantly repress the targeted gene. To improve this, an additional LacI binding site was introduced approximately 100 base pairs upstream of the other LacI binding site, facilitating DNA loop formation by LacI. This loop formation reduces accessibility of the T7 RNAP to the promoter, thereby improving repression efficiency.^33^

TetR is another repressor frequently used for regulating gene expression. Similar to how LacI-regulated genes can be induced with isopropyl β-D-thiogalactoside (IPTG), TetR-regulated genes can be induced by tetracycline, which releases TetR from the operator and allows transcription. TetR has been applied to repress the T7 promoter in CFS and is well-characterized and can achieve a 10-fold repression in CFSs.^46^

While the use of natural transcriptional repressors to build gene circuits has been demonstrated to successfully construct complex circuits such as a 5-node repressilator,^1^ the library of native TFs remains surprisingly limited and constructing GRNs with higher complexity requires a larger library of orthogonal TFs. Methods such as directed evolution of proteins^47^ or de novo design of TFs are promising avenues to expand this regulatory toolbox.^31,48^ Another potential solution involves using programmable modules to build TFs, which will be introduced in the following sections.

### 2.3 Synthetic Zinc Fingers

The zinc-finger is an intensively studied protein motif and the largest TF family. The zinc-finger motif is notably small, the molecular weight of a single C2H2-type zinc-finger domain is only 3 kDa. Researchers developed large libraries of zinc-finger variants, enabling the design of zinc-fingers that can target specific DNA sequences. It was demonstrated two decades ago that zinc-fingers can be engineered to construct artificial TFs, and by either fusing with activation or repression domains the synthetic zinc-finger can function as an activator or repressor.^49^ Zinc-fingers were also applied to gene editing by fusing the zinc-finger domain targeting specific DNA sequences with a nuclease.^50^ An open-source synthetic zinc-finger library named “OPEN” was later published, providing a comprehensive method for engineering zinc-finger arrays.^51^ Online tools are available to assist researchers in designing zinc-finger arrays, enhancing their accessibility.^52^ A recent study introduced a machine learning method to design zinc-finger arrays, showing potential to significantly expand the zinc-finger protein library and facilitate the design of synthetic TFs.^53^ Importantly, for use as GRN components it is often not required to design zinc-finger arrays with a specific sequence specificity as the binding sequence can be easily adapted to the measured zinc-finger specificity.^31^

Building on advances in zinc-finger array engineering, synthetic TFs can be engineered to target a large variety of sequences for building up complex functionality. For instance, Khalil et al. engineered and characterized up to 10 synthetic zinc-finger TFs with high orthogonality, and built transcriptional circuits in yeast.^54^ Furthermore, synthetic zinc-finger TFs were used to characterize clusters of low-affinity binding sites showing that low-affinity binding sites are indeed functional elements that precisely control gene expression *in vivo*.^55^

Synthetic zinc-finger proteins have proven to be valuable tools as repressors in CFSs. A library of 64 zinc fingers was previously characterized using a high-throughput microfluidic method.^31^ These characterized zinc-fingers were subsequently used as transcriptional repressors in CFSs. Zinc-finger binding sites were engineered at the promoter region where native RNAP initiates transcription.^3^ The zinc-finger binds to the target site, inhibiting the interaction between RNAP and the promoter (Figure 2C). Additionally, zinc-fingers were designed to work cooperatively by fusing leucine zipper interaction domains. This cooperativity increases binding affinity and the Hill coefficient, resulting in nonlinearity that can aid in constructing logic gates or gene circuits. With advancements in the *de novo* design of zinc-finger proteins, it is feasible to construct large gene circuits with zinc-finger based regulatory components. The main advantages of zinc-fingers are that they are quite small compared to other programmable regulatory components (9 kDa for a 3 finger DNA binding domain) and that their binding and dissociation rates to DNA are quite fast. The former makes it easier to generate zinc-finger transcription factors using standard cloning, PCR based methods or gene synthesis and allows efficient expression in CFSs. The latter is important if highly dynamic GRNs need to be built.

### 2.4 Transcription Activation-Like Effector (TALE)

Transcription Activation-Like Effector (TALE) is a programmable protein motif that contains a central tandem repeat domain capable of recognizing DNA sequences. Each repeat recognizes a single nucleotide, allowing the TALE to bind to a specific DNA sequence in a modular fashion, which makes it very easy to engineer TALEs with novel binding specificities. Recent research has shown that TALEs can also be utilized as a transcriptional repressor in CFSs.^32^ TALE binding sites were placed downstream or upstream of the T7 promoter respectively (Figure 2D). When the TALE binding site was placed downstream, TALE acted as a roadblock of polymerase during transcription, preventing T7 RNAP from elongation and resulting in a 2-fold repression of transcriptional activity. Conversely, when the TALE binding site was located upstream of the T7 promoter, TALE acted as a repressor that interfered with RNAP interaction with the promoter, which achieved 5-fold repression. The result shows that TALE has the potential to be engineered to target different DNA sequences for constructing a larger orthogonal regulatory network. Nevertheless, TALE proteins are relatively large with a molecular weights of around 100 kDa and their binding kinetics are slow.

### 2.5 STARs

STARs (Small Transcription Activating RNAs) are RNA oligos engineered to activate specific target genes. These genes are positioned downstream of a transcriptional terminator that forms a hairpin structure, which halts transcription. STARs are designed to be complementary to both the terminator sequence and its surrounding regions. In the presence of STARs, the formation of the hairpin structure is disrupted, preventing termination and thereby allowing transcription to continue, leading to the activation of the downstream gene (Figure 2E).^56^ This method has been successfully established in CFSs to construct logic gates.^35^

STARs are programmable transcription regulation components that can be designed based on different terminators and their flanking sequences. Liu et al. constructed a library of orthogonal STARs and provided a method to computationally design STARs,^57^ which is highly beneficial for gene circuit design. Since the mechanism relies solely on RNA without the need for translation, it promises a faster dynamic response compared to protein regulators. This characteristic is advantageous for gene circuits that require rapid response times and can minimize loading effects. However, STARs function only with bacterial RNAP, there is no STAR available for T7 RNAP to our knowledge. Designing STARs for T7 RNAP will be challenging due to the low efficiency of T7 termination,^58^ which will lead to gene expression leakiness.

### 2.6 CRISPR/dCas9

CRISPR/Cas9 has revolutionized gene editing by eliminating the constraints imposed by the sequence specificity of different restriction enzymes, allowing nearly arbitrary sequences to be targeted using designed guide RNAs. The dCas9 mutant form of Cas9, also known as endonuclease-deficient Cas9, has no nuclease activity but retains the ability to bind to target DNA. This property makes dCas9 a versatile repressor that can target various promoters by altering the gRNA sequence. It is widely used in living cells to inhibit transcription.^59^ Beyond cell-based systems, the CRISPR/dCas9 system has also been applied to construct logic gates in CFSs. To incorporate dCas9 into the cell-free lysate protein expression system (Figure 2F), Lehr et al. pre-expressed CRISPR-associated proteins in *E. coli* from which lysates were produced.^36^

Another approach to integrating CRISPR/dCas9 into CFSs is to express the dCas9 protein within the CFS itself by adding the DNA template for the dCas9 protein. However, producing large and complex proteins in a CFS is often somewhat challenging, and dCas9 is a very large protein at 190 kDa. The commercial cell-free expression kit, PURExpress, has successfully produced CRISPR/dCas9, demonstrating the potential of CFSs to produce regulatory components associated with CRISPR to control gene expression.^60^ This result shows that dCas9 protein can be expressed by the PURE system and function as a regulatory component. However, producing dCas9 consumes significant resources, which can cause loading effects, thereby reducing the capacity for implementing larger regulatory networks.

In addition to its use as a repressor, dCas9 can also activate transcription by guiding RNAP to the promoter, known as the CRISPR activation (CRISPRa) system.^61^ Recently, this has been achieved in the *E. coli* lysate system and combined with the inhibitory dCas9 system.^37^ Researchers used dCas9 to bind gRNA with an additional loop, recruiting the *E. coli* SoxS transcription factor, which activates transcription. This combination of programmable activation and inhibition mechanisms with dCas9 enables the design of large-scale gene circuits based on programmable gRNAs. Unlike native transcriptional repressors and activators, dCas9 exhibits very long dwell times on DNA, likely limiting its use to slow acting or responding regulatory systems.

### 2.7 T7 RNAP and Promoter Engineering

T7 RNAP is a widely used RNAP in CFSs, making it essential to develop regulatory mechanisms that control transcription by this polymerase. Fine-tuning promoter strength is the first step in regulating T7 RNAP transcription. A large library of T7 promoter sequence variants has been quantified to assess promoter strength,^62,63^ providing a means to adjust transcription activity. This library spans an activity range over 4 orders of magnitude. Notably, recent research identified a promoter sequence, T7Max, which exhibits stronger promoter strength than the native T7 promoter, expanding the upper limit of the T7 RNAP transcription dynamic range.^64^

**Figure 3:**
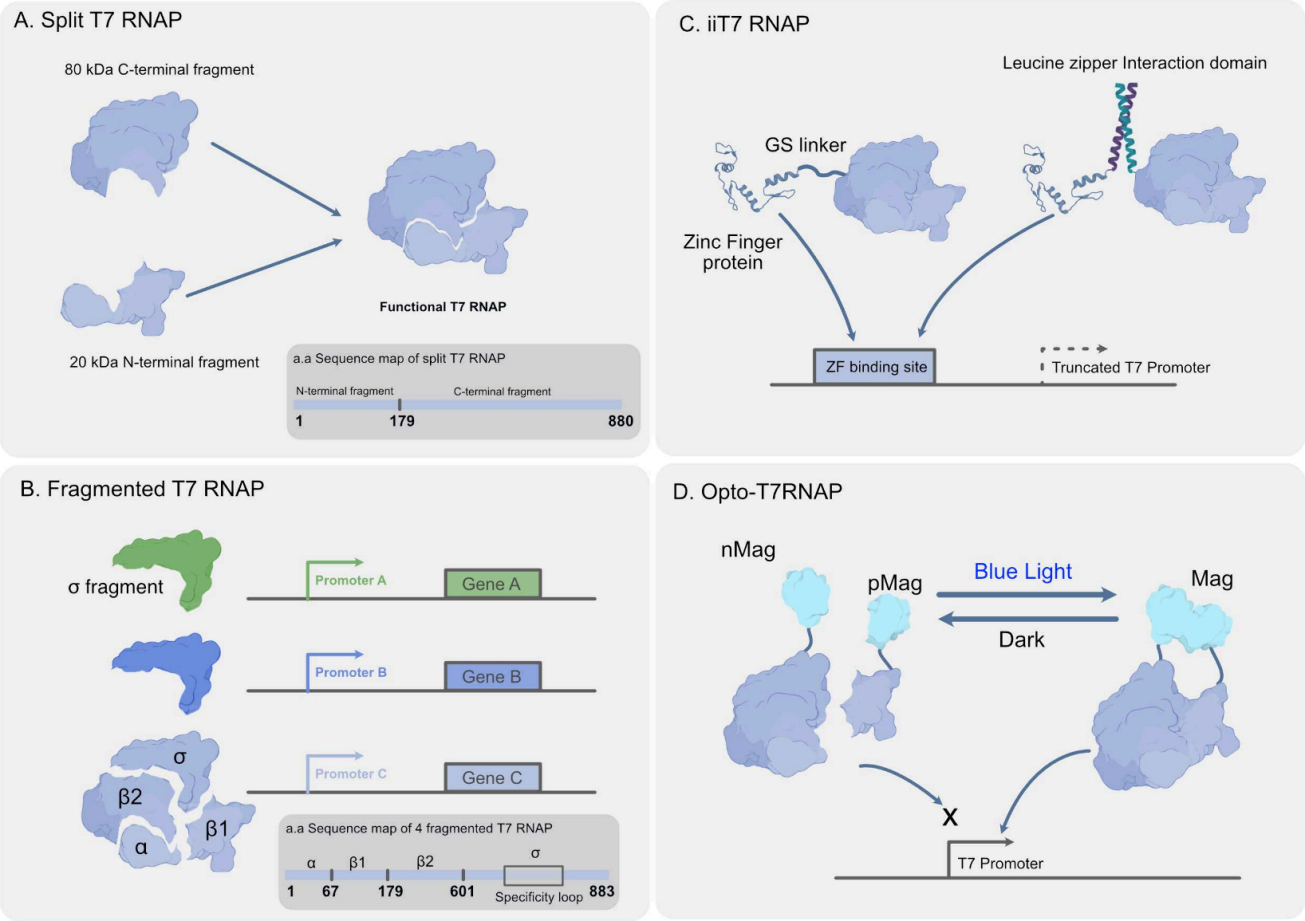
T7 RNAP engineering. (A) Split T7 RNAP consists of two fragments: an 80 kDa C-terminal fragment and a 20 kDa N-terminal fragment. These fragments reassemble to form a functional T7 RNAP.^39^ (B) Fragmented T7 RNAP composed of 4 domains, one domain is responsible for specific DNA binding, and the rest of the domains are responsible for transcription.^40^ (C) iiT7 RNAP is engineered by directly fusing a zinc finger domain with a T7 RNAP or through leucine zipper interactions, allowing targeting of different zinc finger binding sites for initiating transcription. The iiT7 system uses native T7 RNAP in combination with a reduced promoter that is not activated unless an additional zinc-finger binding site is present to recruit T7 RNAP to the promoter.^38^ (D) Opto-T7RNAP involves light-regulated control of T7 RNAP activity. In the presence of blue light, the nMag and pMag proteins will dimerize and transcribe the target gene. The dimerization is reversible, in the absence of blue light, transcription will be inactive.^41^

Split T7 RNAP represents an approach where T7 RNAP is divided into two separate parts that become functional only upon reassembly (Figure 3A).^39^ Building on this concept, Segall-Shapiro et al. developed an advanced version of the split T7 RNAP, further subdividing it into four distinct components (Figure 3B).^40^ One of these components serves as an analog to the native RNAP σ factor, which is essential for initiating transcription at specific promoter sequences. This innovation broadens the regulatory scope of gene expression within a CFS driven by T7 RNAP. The σ factor analog can be exchanged with other analogs to recognize different promoter sequences, thereby activating genes controlled by various promoters. However, *de novo* design of the σ factor analogs poses significant challenges. The four σ factor analogs used by Segall-Shapiro et al. are derived from other phage RNAPs. These analogs lack the programmability characteristic of zinc-finger proteins and do not achieve the efficiency of the original σ analog fragment from T7 RNAP. Recent advancements in protein design hold the potential to create more efficient σ analogs with varying targeting capabilities, thereby improving the regulation of multiple genes in CFS.

A highly programmable T7 RNAP system has recently been developed, utilizing a zinc-finger-T7 RNAP fusion protein, which demonstrated orthogonal regulation of genes in *E. coli* (Figure 3C).^38^ In this system, the T7 promoter is truncated to prevent native T7 RNAP from binding and initiating transcription. A zinc-finger protein, which recognizes a binding site upstream of the truncated promoter, recruits the fusion protein to the promoter, resulting in transcription initiation. Due to the well-characterized nature of zinc-finger proteins and the ease of *de novo* designing novel specificities, this fusion protein can be engineered to recognize synthetic promoters with nearly any sequence. Additionally, a more complex systems was engineered by incorporating a leucine zipper domain. This modification allows different zinc finger proteins to utilize the same T7 RNAP, thereby enhancing system efficiency. The T7 RNAP system fused with leucine zipper is analogous to the bacterial RNAP holoenzyme, with the zinc-finger - leucine zipper fusion being similar to σ factor that guides RNAP to the target promoter. The incorporation of the leucine zipper domain also increased transcription efficiency compared to the direct fusion protein. Moreover, it was demonstrated that gene expression can be repressed by expressing a competing zinc-finger protein that binds to the zinc-finger binding site. This system paves the way for the design of highly orthogonal and programmable transcriptional gene regulatory circuits, offering significant advancements in CFS gene circuit design.

A light-inducible T7 RNAP, called opto-T7 RNAP, was developed by Baumschlager et al. (Figure 3D).^41^ Similar to the split T7 RNAP, opto-T7 RNAPs consist of two parts, each fused to a Magnet protein subunit. These Magnet protein subunits dimerize upon exposure to blue light and reversibly dissociate in its absence. In *E. coli* this system achieved a dynamic range of up to 300-fold upon induction. It is highly tunable, allowing for immediate gene repression when the blue light is switched off. The use of light as a regulatory signal is noninvasive and reversible, providing a clean and rapid method to switch gene expression on or off. Although this system has not yet been applied in CFS, it holds significant potential. Combining this light-inducible system with cell-free technology could enable precise spatiotemporal control of gene expression in synthetic cells.

## 3 Translational Regulation

Translation is driven by the ribosome and various translation factors that facilitate the assembly, elongation, disassembly, and ribosomal recycling processes. The efficiency of translation in CFSs hinges on the activity of the ribosome, the translation factors, and their interactions with mRNA. A key element in this process is the ribosome binding site (RBS), which includes the Shine-Dalgarno (SD) sequence and the upstream TA-rich region. The accessibility of the RBS to the ribosome is paramount for effective translation. The following sections describe regulatory components that modulate the accessibility of the RBS and start codon AUG, thereby controlling translational activity.

### 3.1 Riboswitch

**Figure 4:**
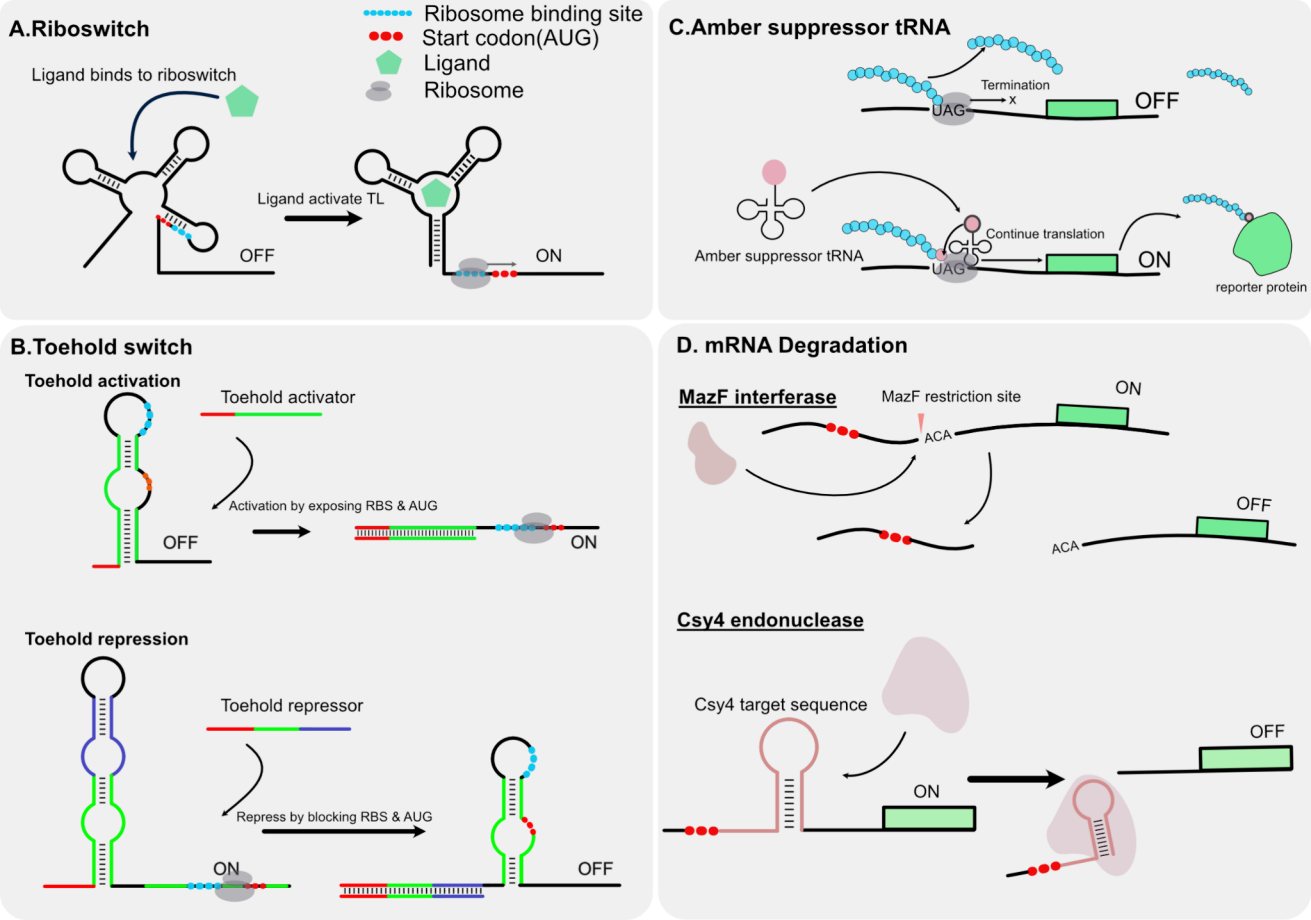
Mechanisms of translational regulatory and RNA degradation components. (A) Riboswitch: A regulatory RNA segment that controls gene expression in response to ligand binding. Ligand binding to RNA induces a conformational change, activating or repressing translation by exposing or occluding the RBS.^42^ (B) Toehold Switch: An engineered RNA switch that can be activated or repressed by complementary RNA oligonucleotides. Activation occurs when the toehold region is bound by the activator RNA, exposing the RBS and start codon to initiate translation. Repression happens when the RBS and start region is blocked by the secondary structure with the complementary RNA.^43,44^ (C) Amber Suppressor tRNA: a tRNA that recognizes amber stop codons (UAG) during translation, allowing the ribosome to bypass the stop signal and continue translation, enabling the expression of the gene.^22,65^ (D) mRNA degradation: It involves specific ribonucleases that degrade targeted RNA sequences. For example, MazF is an endoribonuclease that cleaves mRNA at specific sequences, leading to translation inhibition if the cleaving site is upstream of the targeted gene. Csy4 is another endonuclease that recognizes a specific RNA secondary structure that can be placed upstream of the targeted gene to inhibit translation as well.^36,66^

A riboswitch is an RNA with secondary structure that undergoes conformational changes upon interaction with specific molecules (Figure 4A). This structural change can either obscure or expose the RBS and the translation start codon, thereby inhibiting or activating translation. SELEX (Systematic Evolution of Ligands by Exponential Enrichment) is a method that can be applied to identifying RNA aptamers, that can bind to specific target molecules. Riboswitches can be selected by SELEX for a wide variety of ligands, including ions,^67^ ATP,^68^ and amino acids.^69^ A recent publication also shows that riboswitches can be designed computationally to sense a target protein.^70^ These riboswitches can function as sensors in GRNs, making the system more resource-efficient by eliminating the need for expressing protein sensors. Tabuchi et al. recently reviewed riboswitches that have been applied in CFSs,^42^ including riboswitches that can sense molecules like cGMP, Theophylline, and Dopamine. Compared to their application in living systems, the use of riboswitches in CFSs remains relatively limited. Many riboswitches still need to be tested in CFSs, but they hold great potential as tools for constructing feedback loops or toggle switches, which require the ability to react to molecular cues.

### 3.2 Toehold Switch

Toehold switches regulate translation by modulating the accessibility of the RBS and start codon (Figure 4B). Unlike riboswitches that respond to small molecules, the toehold switch is designed to respond to RNA oligos with specific sequences, thereby altering the secondary structure of the switch. This mechanism does not require sequence complementarity to the RBS or start codon. Instead, it forms a secondary structure with the distal region of the RBS and start codon, offering greater design freedom due to fewer sequence constraints.

The toehold switch can function as both an activator and repressor, depending on the secondary structure change that occurs before and after the binding of the trigger RNA. The accessibility of the start codon can be modulated to be high or low, depending on the sequence design. Due to the flexibility of toehold switches, it is straightforward to create orthogonal regulators that operate independently of one another. Green and Kim et al. demonstrated the construction of complex toehold switches by integrating multiple toehold switches at the 5′ untranslated region (UTR) of the gene of interest. In CFSs, toehold switches have been designed to sense four different inputs and respond accordingly, achieving up to 400-fold repression.^43,44^ This method offers another highly programmable tool for constructing GRNs. Since it is an RNA regulator, the repression and activation dynamics will differ from those of protein regulators, necessitating detailed characterization to build more predictable gene circuits. The unique characteristics of toehold switches, such as their high specificity and flexibility, make them valuable for creating sophisticated and responsive circuits.

### 3.3 Amber suppressor tRNA

An amber suppressor tRNA can be used to regulate gene translation as well (Figure 4C). In this approach, the stop codon (UAG) is placed downstream of the start codon of the target gene. Without the amber suppressor tRNA, translation aborts due to the presence of the stop codon. The presence of the amber suppressor tRNA allows the ribosome to read the stop codon and continue translating the target gene. This mechanism has been successfully applied in CFSs to construct gene circuits.^22,65^ It is particularly useful as a tool to minimize or entirely eliminate leaky expression, which is a common problem in circuit design.

## 4 RNA Degradation

### 4.1 MazF Interferase

Regulating the degradation of mRNA can directly modulate protein expression. Ribonucleases that sequence-specifically degrade mRNA can be used for this purpose. One example is the MazF interferase, which targets single-stranded mRNA containing the ACA sequence and cleaves the 5′ end of the RNA. If the ribosome binding site and start codon are designed to be located upstream of the target gene, MazF interferase can inhibit translation by cleaving this critical region.^66^ To expand the toolbox for degrading RNA containing specific sequences, MazF interferase has been engineered to target other RNA sequences by exchanging the RNA binding loop region.^71^ However, these engineered MazF variants have not yet been tested in CFSs and require further investigation. Specificity may also be an issue, particularly when more complex regulatory topographies are required.

### 4.2 Cys4 Ribonuclease

Csy4 is a CRISPR-associated nuclease that processes gRNA after transcription (Figure 4D). It cleaves transcribed long RNA into separate gRNAs, enabling multiplexed CRISPR/Cas9 regulation. The enzymatic activity of Csy4 is sequence-specific. By placing the Csy4 targeting sequence between the RBS and the target protein gene, the RBS is cleaved in the presence of Csy4, thus inhibiting translation. Previously, Csy4 was utilized in a cell-free lysate system by expressing it in *E. coli* and extracting the lysate from the strain. The Csy4 cleavage site was designed upstream of the RBS and 5′UTR. This demonstrated that Csy4 could effectively cleave the target site and significantly reduce protein expression.^36^ While eukaryotic cells have a large repertoire of RNA degradation tools, such as miRNA-based regulation, RNA degradation strategies in cell-free systems remain limited. However, the toehold switch could offer a potential solution to this limitation, providing an programmable translational regulatory mechanism for PURE or bacterial CFS.

## 5 Protein Degradation

Protein degradation can be useful for creating gene circuits such as pulse generators or reducing the time to reach steady-state protein concentration.^72^ ClpXP has been applied to regulate protein degradation in CFS. It is an essential protease involved in protein degradation, working in conjunction with the SspB protein, which recognizes proteins with a ssrA tag and guides them to ClpXP for targeted degradation. In the PURE system, ClpXP has been shown to lead to an 80% decrease in protein concentration at steady state in cell-free chemostat experiments.^22^ This system was also implemented in all-*E. coli* lysate toolbox 2.0 for gene regulation by Garamella et al.^45^ The ClpXP protein present in *E. coli* lysate was found to be inefficient, so a plasmid with tandem clpP-clpX genes under the P70a promoter was cloned, enabling ClpXP overexpression by the lysate CFS itself. Preincubating the ClpXP plasmid for 1 h showed the highest rate of protein degradation, achieving up to 250 nM per minute, whereas, without preincubation, the maximum degradation rate was 130 nM per minute. The degradation rate of the system can be potentially tuned by preincubation time and clpP-clpX plasmid concentration. However, the ClpXP system requires additional ATP for full functionality, necessitating optimization for implementation in different CFSs.^73^

## 6 External Control Systems

### 6.1 Light-Regulated Gene Expression

Optogenetics is widely used to manipulate living organisms using light, allowing for rapid and precise control of gene expression. Besides the opto-T7 RNAP system introduced above, other examples demonstrated the application of light to control gene expression. One such example is the YF1/FixJ system, an optogenetic tool based on two components from bacteria. FixJ is a transcription factor whose DNA binding affinity is determined by its phosphorylation state. When phosphorylated, FixJ binds to target DNA sequences and activates transcription. YF1 is a kinase that regulates the phosphorylation of FixJ and is sensitive to blue light. Under dark conditions, YF1 autophosphorylates and thereby phosphorylates FixJ. In contrast, under blue light, YF1’s autophosphorylation is inhibited, preventing the phosphorylation of FixJ. Depending on the design of the system, FixJ can be engineered to either activate or repress the expression of the target gene. This system demonstrated 5-fold activation and 3-fold repression of gene expression.^74^

Other light-regulated components, such as photocaged DNA, can be triggered by blue or UV light to control gene expression. In these studies, the T7 promoter is inhibited by photocleavable molecules that are sensitive to blue or UV light.^75,76^ Additionally, reversible chemically modified DNA components sensitive to light have been developed. For instance, Kamiya et al. modified DNA templates for protein expression using azobenzene derivatives, which can be isomerized by visible or UV light. When the azobenzene-modified DNA is in the “on” state, the regulated gene is activated.^77^

### 6.2 Temperature-Regulated Gene Expression

CFSs can be run over a relatively wide temperature range of 20–40 °C, allowing for the design of gene regulation methods based on varying reaction temperatures within this range. Yang et al. developed a temperature-based gene expression regulation method using the heat-sensitive characteristic of the CI repressor.^34^ Below 30 °C, the CI repressor forms a dimer and strongly binds to the targeted binding site, repressing transcription. However, below 37 °C, the CI repressor cannot form a dimer and is released from the DNA binding site. By placing the CI binding site close to the promoter region, transcription can be easily induced by increasing the temperature. At 37 °C gene expression can increase up to 147-fold compared to noninduced conditions. However, temperature variations affect the entire CFS, so further optimization is needed to integrate this mechanism effectively.

## 7 Examples of Cell-Free Genetic Networks

### 7.1 Logic Gates

Logic gates are fundamental components in computation, and their principles can be applied to the design of gene circuits to program gene expression. These gates process input information to produce particular outputs, making them essential building blocks for constructing complex functions within GRNs.

**Figure 5:**
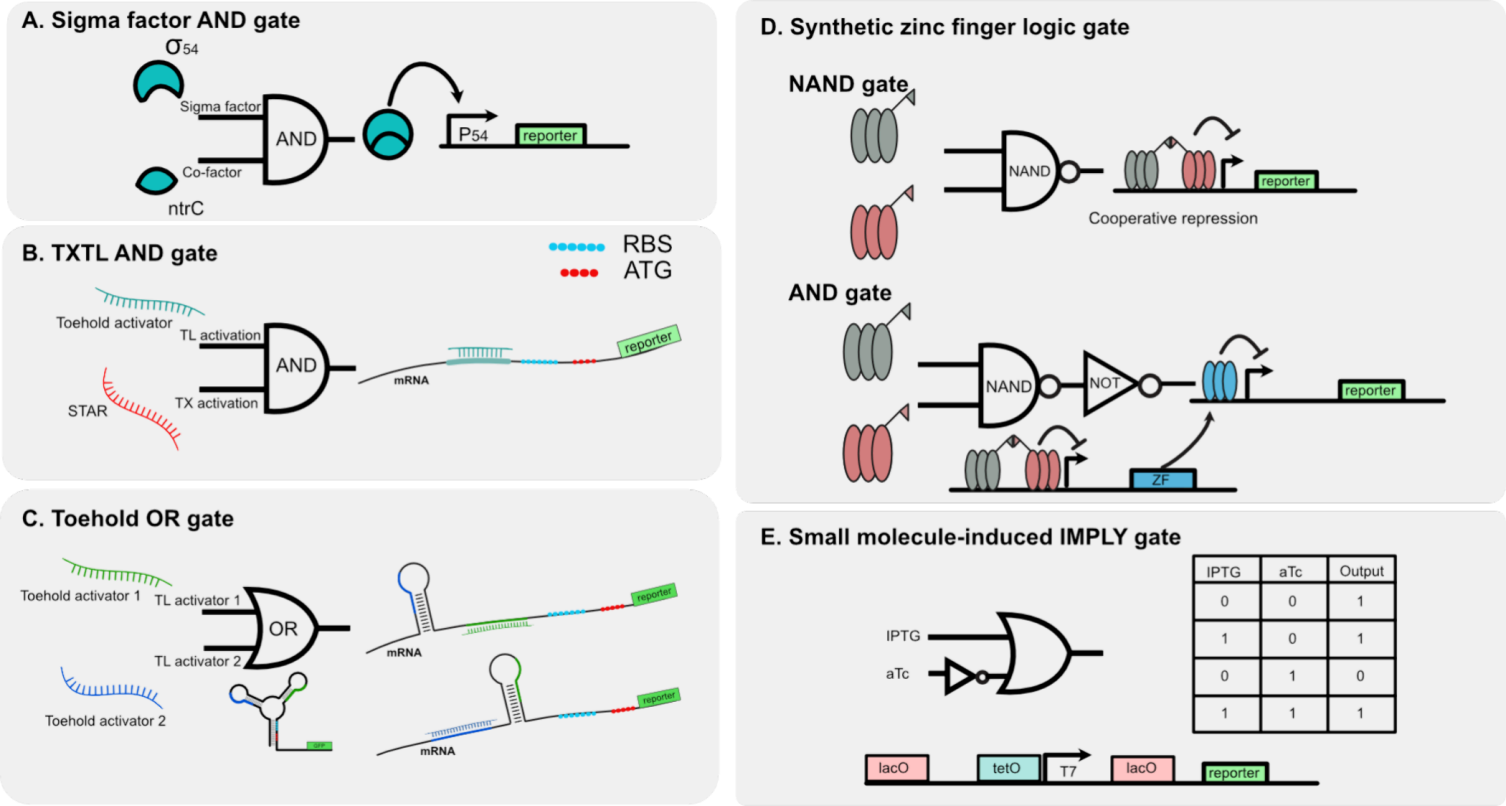
Cell-free logic gates. (A) AND gate built from sigma factor and its cofactor, the targeted gene will only be expressed when both factors are presented.^78^ (B) AND gate built with STAR and toehold activator RNA, STAR will activate transcription and the toehold will activate translation of the targeted gene.^35^ (C) OR gate built with two toehold activator RNAs, each toehold activator alone can expose the RBS and start codon of the targeted gene.^79^ (D) IMPLY gate constructed using inducible repressors LacI and tetR. LacI represses the target promoter by forming a DNA loop, inhibiting gene expression. TetR disrupts this loop, thereby activates the gene. When aTc is added, it binds to tetR, causing dissociation from the operator and allowing the loop to reform, thereby repressing the target gene again.^33^ (E) NAND, AND gate built with zinc-fingers. Two synthetic zinc-fingers bind to the promoter region cooperatively, forming a NAND gate. AND gate is achieved by placing a NOT gate consisting of a single-zinc finger downstream of the NAND gate.^3^

In CFSs, logic gates have been developed using different gene regulation methods. For instance, an AND gate in gene circuits can be constructed using a sigma factor and its cofactor (Figure 5A), where the target genes are activated only when both proteins are present.^78^ This basic design ensures that gene expression occurs only under specific conditions where multiple signals are detected. In addition to the transcriptional-regulated AND gate, the combination of transcriptional and translational regulators is applied to construct the AND gate. Lehr et al. utilized STARs as a transcriptional activator and toehold as a translational activator to activate a gene^35^ (Figure 5B). This approach offers a wide dynamic range with a high fold-change. Additionally, the use of RNA oligos as activators on both transcription and translation eliminates the necessity for protein regulators, simplifying the design and enhancing modularity. Ma et al. develop a toehold regulatory mechanism so that regulated genes can be activated by only one of the two activator sequences independently (Figure 5C), thus achieving the OR gate function.^79^ Toehold switches can also achieve a complex AND gate with up to 4 inputs.^44^

An IMPLY gate was constructed by combining two inducible repressors. The two inputs of the gate are small molecules that detach the repressors from DNA^33^ (Figure 5D). The LacI repressor can bind to two Lac operators, forming a loop that represses transcription. This loop formation is disrupted by the presence of the tetR repressor, which binds to the tet operator. When aTC is added to the reaction, the tetR repressor no longer affects binding of LacI, thereby inhibiting transcription. Although the IMPLY gate is not as commonly used as AND, OR, or NOT gates in circuit construction, it is known for its ability to minimize circuit design.

Zinc-finger proteins offer another versatile approach for building logic gates due to their programmable characteristics. Previous studies have extensively characterized the affinity and specificity of various zinc-finger protein variants, making them reliable components for constructing logic gates.^3^ Swank et al. employed zinc-finger proteins to create various logic gates, including AND and NAND gates (Figure 5E). For instance, an AND gate can be constructed by fusing a dimerization domain to two individual zinc fingers, enabling them to work cooperatively to repress gene expression. When both zinc-fingers are present, the regulated gene is strongly repressed. This design was extended to build a NAND gate by combining a NOT and AND gate. Given that the NAND gate is functionally complete, if there are enough orthogonal zinc finger proteins available, it can theoretically be used to construct any other logic gate.

### 7.2 Oscillators

Oscillators are fundamental modules in electrical systems and also serve as a central regulatory function in biological systems, including somitogenesis and circadian clocks.^80,81^ In 2000, the first synthetic gene network capable of creating oscillations in *E. coli* was successfully developed.^12^ However, achieving oscillatory networks in cell-free reactions is challenging since the reactions are typically conducted in batch mode. Continuous feeding and dilution were required to maintain oscillations in a CFS.

To address this challenge, the first genetic oscillator was successfully implemented using a microfluidic chemostat.^22^ The circuit is based on positive feedback and delayed negative feedback, utilizing T7 RNAP, TetR repressor, and Amber suppressor tRNA. Another example of a cell-free oscillator was achieved by Tayar et al. using a microfluidic device combined with the DNA nano brush technique.^82^ A more complex design of the circuits was later constructed.^23^

**Figure 6:**
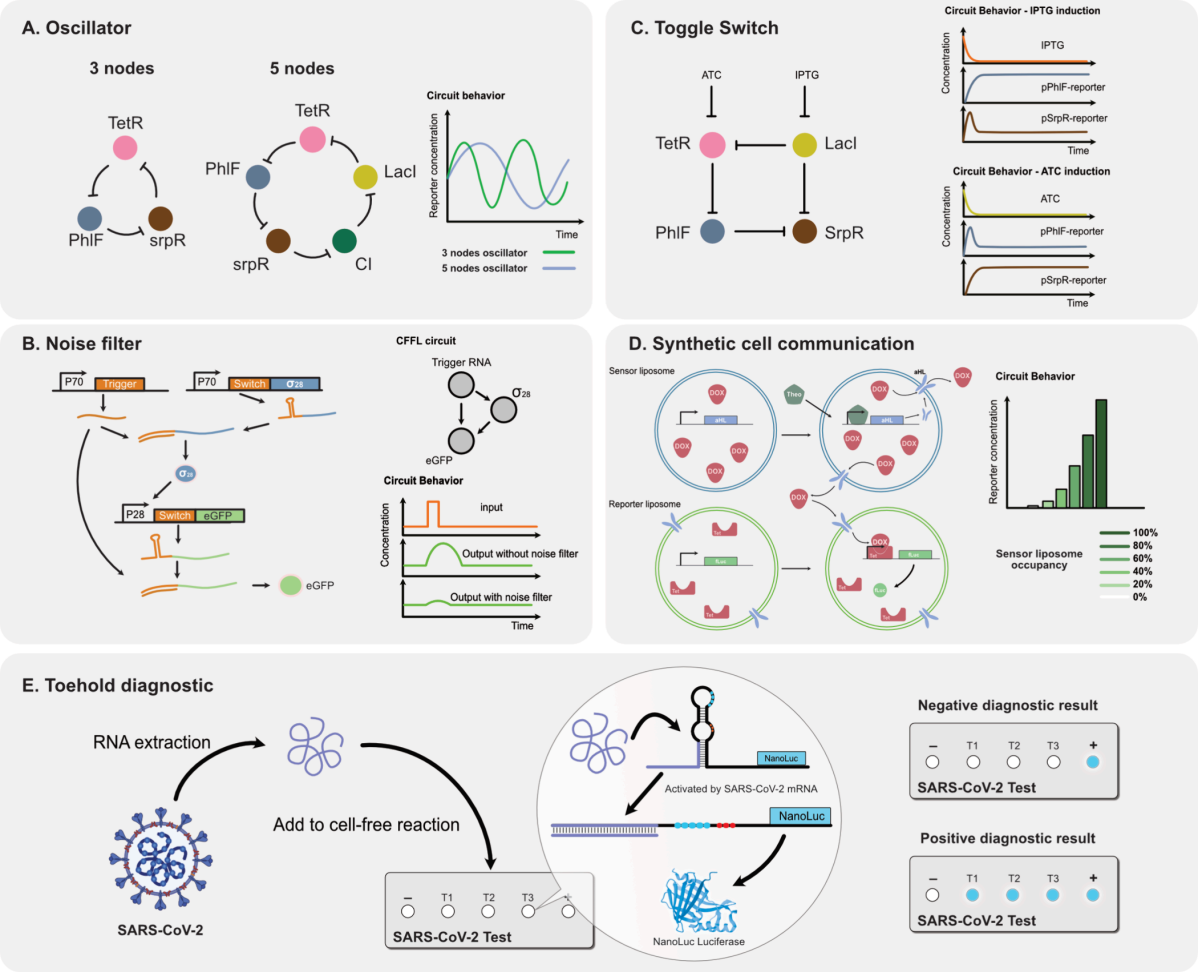
Cell-free gene circuits. (A) 3-node and 5-node oscillator circuits are constructed using natural repressors. The 5-node oscillator exhibits a longer oscillation period compared to the 3-node oscillator.^1^ (B) CFFL noise filter circuit is composed of a toehold activator and sigma factor. Both components must be present to activate the target gene, providing a mechanism for reducing gene expression noise.^28^ (C) The toggle switch circuit is composed of four natural repressors. TetR and LacI can be induced by inducers, thereby changing the state of the switch. After induction, the circuit will stably maintain the same state without supply of inducer.^1^ (D) The synthetic cell communication circuit is composed of the sensor liposome and the reporter liposome. The sensor liposome can sense theophylline and activate expression of an aHL membrane transporter protein. This transporter enables doxycycline to enter the reporter liposome, thereby activating gene expression within it.^29^ (E) The SARS-CoV-2 diagnostic circuit is based on the toehold regulatory mechanism. The toehold structure is designed to be complementary to the viral RNA sequence, and upon binding, it exposes the ribosome binding site (RBS) and start codon, leading to the translation of the reporter protein, which indicates the presence of the virus.^26^

The 3-node repressilator oscillator first developed in 2000 was later also implemented in a CFS.^1^ The original regulatory components including TetR, CI and LacI repressors were applied to construct the repressilator circuit and other 3-node oscillators using different repressors were also built. Niederholtmeyer et al. also built the first 5-node repressilator using the PhIF, SrpR, BetI and QacR repressors (Figure 6A). It was also demonstrated that oscillators that were engineered *in vitro* could be functionally transplanted to an *in vivo* system. Surprisingly these novel 3-node repressilators functioned much more robustly *in vivo* than the original repressilator built by Elowitz et al. indicating that the repressilator network topology can give rise to robust oscillations. Yelleswarapu et al. later constructed a gene circuit composed of two oscillators and observed the circuit behavior of coupling two oscillators together.^83^

The advancement of CFS oscillator circuits with microfluidic devices demonstrates the capability to run complex gene circuits *in vitro*. While current CFS oscillator circuits are built from natural repressors, the potential exists to use programmable regulators to further extend the functionality of these circuits.

### 7.3 Noise Filter

Most biological reactions are stochastic and noisy. If a system is too sensitive, minor perturbations can greatly influence its performance. A coherent feed-forward loop (CFFL) is a circuit that acts as a noise filter. In this circuit, an upstream gene directly and indirectly activates a target gene. The regulated gene can only be expressed when both activation signals are present, and the time delay caused by the indirect activation buffers against perturbations. Pieters et al. attempted to build a CFFL circuit in the *E. coli* lysate system (Figure 6B).^28^ They constructed the circuit using toehold switches and sigma factors. A common toehold trigger RNA sequence acts as an activator for the expression of the sigma factor and reporter, both are regulated by the toehold switch. They found that the CFFL circuit they built exhibited background suppression behavior. However, the noise-filtering behavior was not significant experimentally. They simulated the system to search for optimal parameters that could achieve the noise filter function. It remains to be seen if the designed circuit can achieve effective noise filtering. A recent publication demonstrated another noise filtering method, the circuit express MazF nuclease that is regulated under T7 promoter, which creates a negative autoregulation to other genes regulate by T7 promoter. The design can decrease the noise of CFS by more than 2 folds.^84^

### 7.4 Toggle Switch

The toggle switch is a fundamental component that allows a system to change its state in response to internal or external signals. This functionality is crucial for building complex behaviors such as synthetic cell differentiation or memory. The first synthetic toggle switch gene circuit was constructed in 2000 by Gardner et al., who successfully switched the expression of fluorescent proteins using IPTG and temperature in *E. coli*.^11^ To construct a toggle switch circuit, two main features are required. First, the system needs to remain stable in one state and should not change its state spontaneously. Second, the circuit must be responsive to a signal that can switch the system from one state to another. In our laboratory, we previously developed a toggle switch in CFS by utilizing four repressors, two of which are inducible (Figure 6C). This toggle switch was implemented in a cell-free chemostat reaction using a microfluidic device. Initially, IPTG or aTC is introduced to the reaction to set the initial state of the circuit. Once the state was established it remained unchanged throughout the reaction without the need for continuous inducer presence.^1^ Toggle switches have also been achieved using RNA regulators.^85^ In this system, two RNAPs are designated to transcribe RNA aptamers that mutually inhibit each other, resulting in a stable state. The inducer used in this work is an additional RNA aptamer added to the reaction, which can alter the system’s state. These developments highlight the versatility of toggle switch designs in synthetic biology, offering robust and stable control over gene expression states in both protein and RNA-based systems.

### 7.5 Synthetic Cell Communication

Cell communication is an essential requirement for building sophisticated multicellular systems. To achieve communication between encapsulated regulatory circuits in synthetic cells and mimic a multicellular system, signal-releasing and signal-receiving components are essential. Both components require corresponding regulatory elements to respond to target molecules. Several studies successfully created synthetic communication between artificial cells.

Adamala et al. demonstrated communication between synthetic cells composed of lipid membranes and encapsulated cell-free reactions (Figure 6D).^29^ They designed a communication system involving membrane-permeable (theophylline) and nonpermeable (doxycycline) molecules, along with a membrane pore protein (aHL). Their findings show that the gene responsible for expressing the aHL pore protein in the sensor liposome can be activated by theophylline, leading to the diffusion of doxycycline into the reporter liposome. By varying the amount of sensor liposome present, they observed that the expression of a fluorescent protein in the reporter liposome was directly influenced, demonstrating signal transmission from the sensing liposome to the reporter liposome.

Another experiment achieved synthetic cell communication through direct contact. The researchers encapsulated the CFS system in a lipid membrane containing aHL pore proteins. Signaling molecules such as C6-HSL and arabinose could traverse the membrane or membrane pores.^30^ The artificial cells were physically attached to one another, allowing passive diffusion of signaling molecules between them. These molecules acted as activators, triggering the expression of regulated genes and producing fluorescent proteins. The researchers observed morphogen-like behavior in their synthetic multicellular system. They also coupled the system with more complex networks, such as an incoherent feed-forward loop, capable of generating signal pulses.

These achievements demonstrate the feasibility of compartmentalizing different cell-free gene circuits and connecting them for signal propagation. This represents a significant advancement in developing multicellular synthetic cell communication systems by using gene regulatory molecules as signals.

### 7.6 Diagnostic Circuits

CFSs can be stored stably at room temperature after lyophilization. The lyophilized CFS can then be activated by dissolving it in an aqueous solution.^86,87^ This approach provides a cost-effective and convenient tool for diagnostics, eliminating the need for a cold chain for distribution. CFSs can be rapidly designed to detect various target substrates, such as small molecules, nucleic acids, or antigens.^88^ These characteristics make CFSs a potentially valuable tool for on-site and resource-limited diagnostic applications. CFSs have also been used to detect viruses including Noroviruses^27^ and SARS-CoV-2^26^ (Figure 6E). Furthermore, the integration of amplification circuits, such as those involving integrase expression, has enhanced the sensitivity of CFS-based diagnostic platforms, particularly when combined with toehold switch technology.^89^ In addition to viral detection, CFSs have been applied to environmental monitoring, such as the detection of heavy metals in water.^25^ Vezeau et al. have further advanced this technology by developing a computational pipeline for designing riboswitches capable of sensing specific proteins. Their system has been validated for the detection of the MS2 phage coat protein, human interleukin-32γ, and monomeric C-reactive protein.^70^

Some biomolecules can be detected with TFs, for example benzoic acid can activate BenR transcription factor then activate P_Ben_ promoter,^90^ enabling the system to detect benzoic acid. Although there are limitations on the biomolecules that can be sensed by TFs, the current library of detectable biomolecules can be expanded by incorporating enzymes that convert nondetectable biomolecules into detectable ones. A computer-assisted pathway design app “SensiPath” was built to help users design metabolic pathways.^91^ The software provides the necessary enzymes required for converting the target molecules, and these enzymes can be directly supplemented into or coexpressed with the CFS. This approach has been successfully employed to detect hippuric acid and cocaine by enzymatically converting them into benzoic acid using HipO and CocE enzymes, respectively. Recently, a high-throughput, automated workflow utilizing a sonic liquid handler was introduced, capable of screening over 3,682 cell-free reactions under varying conditions to characterize transcription factors. This system offers a powerful platform for developing molecule sensors in CFSs.^92^ Two transcription factor libraries from this study demonstrated detection capabilities for mercury (Hg) and cadmium (Cd), highlighting the potential for rapidly expanding TF-based sensing applications with this pipeline.

CFS can be encapsulated in an artificial membrane to create artificial cells. This compartmentalization provides a stable and optimal environment for protein expression. Boyd et al.^93^ have reviewed artificial cell-based biosensors, including Histamine,^94^ IPTG^29^ or physical signals such as mechanical force.^95^ Additionally, Boyd et al. demonstrated fluoride detection using encapsulated CFS.^96^ These examples demonstrate the utility of regulatory components in constructing diagnostic tools and highlight the potential to create circuits using these components.

## 8 Summary and Outlook

One of the goals of synthetic biology is to build functional biological systems that achieve specific functions. Cell-free synthetic biology offers the potential to rapidly design, build and test new biological systems since components can be directly added and implemented in CFSs without the need for time-consuming cloning and transformation steps. CFSs can also be miniaturized into nanoliter reactions, facilitating high-throughput experiments.^97,98^

Several areas in synthetic biology will benefit from developing novel cell-free gene regulatory components. For example, metabolic engineering requires multiple proteins to be present in the reaction, and the ratio of these proteins might significantly influence the system’s efficiency. With gene regulatory components, different enzymes in the system can in principle be controlled within optimal ranges, maintaining maximum efficiency. Advancements in regulatory components will also facilitate the development of artificial cells. Integrating control systems in artificial cells will be crucial as their complexity increases.^7^ For drug delivery using synthetic cells, the regulatory network can provide computational capabilities to ensure the drug is released only under specific conditions and dosed correctly.^99^

With the increasing push toward more complex regulatory networks, there is a high demand for *de novo* designed regulatory components. Most regulatory components used in synthetic biology are native components, such as LacI and TetR repressors. However, only a surprisingly small number of these native components are regularly used and have been characterized in detail. Emerging programmable components for CFSs include RNA-based regulators like toeholds and STARs. CRISPR/dCas9 has also been applied in CFSs, and zinc-finger proteins can act as transcriptional regulators. These programmable methods open possibilities for building large GRNs.

As circuit design progresses, there will be an increasing need for characterizing and modeling these programmable components as well. Models of the PURE system already exist, forming the basis for rational design of this system.^15−17,100^ However, most computational models are coarse-grained and do not include detailed parameters such as resource availability and allocation. Factors like RNA secondary structure, codon usage, and GC content strongly influence protein expression.^101^ Understanding the relationships between these parameters and protein expression might require large data sets. Besides modeling the core functions of CFSs, incorporating gene regulatory components into the models^102^ will further improve our ability to engineer complex, integrated biochemical systems.

The cell-free community will continue to expand and better characterize the cell-free regulatory toolbox, enabling robust design of novel gene circuits with increased functionalities. Combined with improved and comprehensive computational tools this will facilitate the engineering of complex biochemical systems and we therefore expect significant advancements and a slew of novel CFS applications in the near future.
